# A modified Delphi study to identify which items should be evaluated in shoulder instability research: a first step in developing a core outcome set

**DOI:** 10.1016/j.jseint.2023.06.012

**Published:** 2023-07-14

**Authors:** Lukas P.E. Verweij, Inger N. Sierevelt, David N. Baden, Robert Jan Derksen, Henk-Jan van der Woude, Karin M.C. Hekman, Michel P.J. van den Bekerom, the SINC study group, M. van den Borne, J.A. van der Linde, D.F.P. van Deurzen, O.A.J. van der Meijden, T.D.W. Alta, B. Muller, S. Floor, R.N. Wessel, A. van Noort, B.W. Kooistra, T. Gosens, Y.V. Kleinlugtenbelt, T.D. Berendes, H.C. van der Veen, C. Visser, C.L. van den Brand, A.M.L. Wildevuur-Houthoff, A. Wei, R. Verbeek, D.G. Barten, R.J.C.G. Verdonschot, T. Boeije, F. Roodheuvel, M.A. Huis in ’t Veld, E. Röttger, M. Versteegen, D. Douma, K. Azijli – Abdellaoui, L. Walraven, R. Boden, N. Sluijter, M.L. van Gastel, W. van den Berg, P. Jansen-Oskam, I.S. Haas, H. Nes, G. Koel, B. Hessel, D. Heijblok, I.M. Husen, M. Numan, F. Boon

**Affiliations:** a Amsterdam UMC, Location AMC, University of Amsterdam, Department of Orthopedic Surgery and Sports Medicine, Amsterdam, The Netherlands; b Amsterdam Movement Sciences, Musculoskeletal Health Program, Amsterdam, The Netherlands; c Amsterdam Shoulder and Elbow Center of Expertise (ASECE), Amsterdam, The Netherlands; d Xpert Clinics, Department of Orthopedic Surgery, Amsterdam, The Netherlands; e Spaarnegasthuis Academy, Orthopedic Department, Hoofddorp, The Netherlands; f Emergency Department, Diakonessenhuis, Utrecht, The Netherlands; g Department of Trauma Surgery, Zaandam Medical Center, Zaandam, The Netherlands; h Department of Radiology, OLVG, Amsterdam, The Netherlands; i Shoulder Center IBC Amstelland, Amstelveen, The Netherlands; j Department of Orthopedic Surgery, Medical Center Jan van Goyen, Amsterdam, The Netherlands; k Shoulder and Elbow Unit, Joint Research, Department of Orthopedic Surgery, OLVG, Amsterdam, The Netherlands; l Department of Human Movement Sciences, Faculty of Behavioural and Movement Sciences, Vrije Universiteit Amsterdam, Amsterdam, The Netherlands

**Keywords:** Core outcome set, Delphi, Consensus, Shoulder, Instability, Dislocation, Outcome

## Abstract

**Background:**

The aim of this study was to identify items that healthcare providers and/or patients consider important to include in a questionnaire for clinical trials and cohort studies in shoulder instability research. This could serve as a basis to develop a core outcome set for shoulder instability research.

**Methods:**

Healthcare providers and patients were included in a panel for a modified Delphi consensus study. The study consisted of three rounds, comprising (1) identifying items, (2) rating the importance of the items, and (3) rating the importance again after seeing a summary of the results of round two. Importance was rated on a 9-point Likert scale. Consensus was defined as ≥ 80% of the panel giving a score of 7 or higher.

**Results:**

In total, 44 healthcare providers and 30 patients completed all three rounds. Round one identified 54 items. After round three, the panel reached a consensus on 11 items that should be included in a questionnaire, comprising re-dislocation (99%), instable feeling of the shoulder (96%), limitations during sport (93%), patient satisfaction with the shoulder (93%), fear/anxiety for re-dislocation (91%), range of motion (88%), return to old level of functioning (85%), performing daily activities (85%), return to sport (82%), return to work (82%), and trusting the shoulder (81%).

**Conclusion:**

Healthcare providers and patients reached a consensus on 11 items that should be included in a questionnaire for shoulder instability research. These items can facilitate design and development of future clinical trials and form the basis for the development of a core outcome set.

Patient-reported outcome measures (PROMs) can be used to measure outcomes such as patient’s health status, physical ability, or quality of life. PROM can be measured through a questionnaire that is filled out by the patient individually or assisted by a physician and is often used in orthopedic research.^26^ There has been a paradigm shift toward using more PROM over the past years, as they are believed to be equally important to traditional objective clinical outcomes.^2^^,^^15^^,^^24^ Many PROM tools have been developed that combine questions to get insight into comprehensive outcomes. An example is the Western Ontario Shoulder Instability Index (WOSI), which is a validated instability-specific PROM tool to measure clinical improvement.^14^ A systematic review by Whittle et al identified 28 PROM tools used in shoulder instability research of which some are instability-specific and most are shoulder-specific.^26^ There is no consensus on which outcomes should be used in shoulder instability research and, despite the large number of available PROM tools, new tools are still being developed and it is unclear which PROM tools should be used to measure outcomes in shoulder instability research.^8^^,^^20^^,^^21^^,^^26^

Due to high variety in choices of outcomes in clinical trials, comparing results of clinical trials or pooling individual patient data in meta-analyses can be challenging.^4^^,^^28^ Furthermore, patients are often not included in the design of trials or cohort studies.^4^ A focus group study by van Iersel et al showed that fear of (recurrent) dislocation was an important theme for patients and this is not a common outcome in shoulder instability research.^23^ To provide homogenous data and facilitate meta-analyses of individual patient data, a consensus on which outcomes should be used in shoulder instability research that is based on the opinion of both healthcare providers and patients needs to be reached. The Core Outcome Measures in Effectiveness Trials (COMET) initiative raises awareness for current problems with outcomes in scientific research and encourages the development and uptake of core outcome sets (COS). They promote patient and public involvement in the development of these sets. A COS is a set of agreed standardized outcomes that should be measured and reported in all trials for a specific clinical field.^28^ During the development of a COS, it is agreed *what* should be measured and reported and *how* this should be measured. To facilitate the development of a COS, the COMET initiative has created a handbook with a step-by-step guide.^28^

This study focused on the “what” and aimed to identify which items should be evaluated in a questionnaire used in shoulder instability research. Reaching a consensus can be a challenging process. A Delphi study, which is named after the most visited oracle in classical antiquity, is a well-accepted iterated method to assemble experts and reach a consensus for a controversial topic.^11^^,^^12^ The Delphi study design is structured and comprises multiple anonymous rounds in which the experts can share their opinion. Multiple rounds allow the participating experts to reassess their initial judgment based on the anonymous feedback that is provided through the opinion of other experts in previous rounds. Thus, facilitating reaching unbiased unanimous consensus. The aim of this study was to identify items that healthcare providers and/or patients consider important to include in a questionnaire for clinical trials and cohort studies in shoulder instability research. This could serve as a basis to develop a COS for shoulder instability research. To help identify the outcomes, Macefield et al proposed a method to develop comprehensive items instead of individual PROM tools or questions.^16^ The Delphi design has been used successfully in previous studies to identify items for a COS in pancreatic cancer research.^9^^,^^24^

## Methods

The medical ethics commission NedMec Utrecht (METC NedMec) approved the study design and declared that a thorough ethical evaluation under the Dutch law of scientific medical research was not required (registration number: 22/520). The recommendations for methodologic criteria and reporting for Delphi studies by Diamond et al and Hohmann et al were used.^5^^,^^11^^,^^12^ A modified Delphi design was used, which adds a literature review to the first “open-ended questionnaire” round.^11^^,^^12^ The lead author (L.P.E.V.) served as liaison and created the questionnaires based on the responses. The liaison handled all communication and did not participate in the study to prevent bias in the analyses. All questionnaires were sent using Castor EDC, which is an online tool to send questionnaires and collect data. The Delphi study consisted of three rounds, comprising (1) identifying items, (2) rating the importance of the items, and (3) rating the importance of the items again after seeing a summary of the results of round two. All patients provided consent for participation.

### Assembling the expert panel

The term “expert” is defined as an individual that has relevant knowledge and experience in the field.^12^^,^^13^ Both healthcare providers and patients were included in the expert panel. Healthcare providers in emergency medicine, physiotherapy, and shoulder specialists in orthopedic/trauma surgery who are active in treatment of shoulder dislocations were asked to participate. The network of the authors was used to find suitable candidates for participating in the Delphi study. Recruitment of the healthcare providers was achieved through the network of Dutch medical professional associations, such as the Dutch Association for Emergency Care Doctors (NVSHA), Dutch Shoulder Network for physical therapists, Dutch Orthopedic Association (NOV), and Dutch Association for Trauma Surgery (NVT). They were sent an email to participate. Patients were recruited from a physiotherapy clinic in Amsterdam and should have experienced at least 1 anterior shoulder dislocation to be able to participate. A consensus on how many experts should be included in a Delphi study has yet to be reached, as this could vary based on the topic. A previous study suggested to include 10-15 subjects if their background is homogenous.^13^ We considered specialists in each specific field to be a relatively homogenous group as they share the same goal when treating a patient. Patients were considered to be more heterogeneous because they might not share the same goal while being treated. In addition, patients undergo different treatment regimens. Therefore, we aimed to include at least 10 participants per specialty (minimum of 40) with both academic and clinic backgrounds and at least 30 patients following nonoperative treatment or operative treatment. By including this number of participants, it was believed that every group was adequately represented.

### First round

The first round identified items through a literature review in combination with two open-ended questions. The lead author (L.P.E.V.) performed a literature search in the PubMed database to find systematic reviews about PROM in shoulder instability research using keywords such as “patient-reported,” “PRO,” and “shoulder instability.” All PROMs were extracted from the reviews and changed to comprehensive items according to the method by Macefield et al by the lead author.^16^ For example, the question *“How much difficulty do you have sleeping because of your shoulder?”* of the WOSI questionnaire would fall under the item “sleep.”^14^ Subsequently, the items were checked by the last author (M.P.J.B.) for accuracy. All items were classified according to the International Classification of Functioning, Disability and Health of the World Health Organization, with the additional classifications “health-related quality of life” and “patient experience” according to Cella et al.^3^^,^^27^ If disagreement arose regarding any of the descriptions, the study team discussed a more appropriate use of words. A list of the items identified through the literature review was sent to the panel, whom were asked the open-ended questions (1) *“can you think of any additional items that could be added to this list?”* and (2) *“would you phrase any of the items presented here differently?”*. This resulted in a final list of items. Furthermore, baseline characteristics regarding occupation and experience of the healthcare providers and type of dislocation and treatment of the patients were acquired.

### Second and third round

During the second round, the panel was asked to rate the importance of the items using a 9-point Likert scale (1 being not important and 9 being very important) according to the question “*How important do you think it is that this item is addressed in a questionnaire to evaluate patients that experienced a shoulder dislocation?”*.^9^^,^^12^ The results of round two were summarized in histograms that showed how many participants rated the items with a 7 or higher. This summary was presented to the panel before they entered round three. After seeing the summary of round two, the panel rated the items again in round three. Consensus was defined as ≥ 80% of the panel giving a score between either 1 and 3 (item should never be used in a questionnaire) or 7 and 9 (item should always be used in a questionnaire) in round three. When ≥ 90% of the panel gave these scores, it was defined as a strong consensus.

### Data collection and analysis

Baseline demographic data were presented as average and standard deviation or median and interquartile range according to their distribution. For patients, these included age, sex, education level, amount of experienced dislocations, sports level, work, and if they underwent surgical treatment. For healthcare providers, these included age, gender, medical specialty, and years of experience. The categorical variable ‘consensus’ was expressed as absolute numbers (percentages). Partial deletion was applied to account for missing data if it was present. Data were analyzed using Excel (Microsoft Corp., Redmond, WA, USA).

## Results

### Characteristics of the panel

A total of 59 healthcare providers and 50 patients were contacted by email and 47 (80%) healthcare providers and 34 (68%) patients agreed to participate in this modified Delphi study between the 1st of June and 31st of October 2022. The three rounds were completed by 44 healthcare providers (94%) and 30 (88%) patients (Table I).

**Table I:** Characteristics of the panel.

Patients (n = 30)	
Age, *average (SD)*	31 (11.7)
Male sex, *n* (*%*)	14 (47)
Education level, *n* (*%*)	
Elementary school	2 (7)
High school	1 (3)
Higher education	6 (20)
University	22 (73)
Dislocations experienced, *median (IQR)*	5 (8)
Active in sports, *n* (*%*)	26 (93)
Sports level, *n* (*%*)	
Recreational	17 (65)
Competitive	8 (31)
Professional	1 (4)
Work, *n* (*%*)	27 (90)
Surgery, *n* (*%*)	9 (30)
Arthroscopic labral repair	7 (23)
Glenoid augmentation	2 (7)

Healthcare providers (n = 44)	
Age, *average (SD)*	42 (8.3)
Male sex, *n* (*%*)	31 (68)
Specialism, *n* (*%*)	
Orthopedic/trauma surgery	17 (38)
Emergency care medicine	15 (34)
Physiotherapy	12 (27)
Years of experience, *median (IQR)*	12 (7)
Years of experience as shoulder specialist, *median (IQR)*	10 (7.5)

*SD*, standard deviation; *IQR*, interquartile range.

### First round: item identification

**Figure 1:**
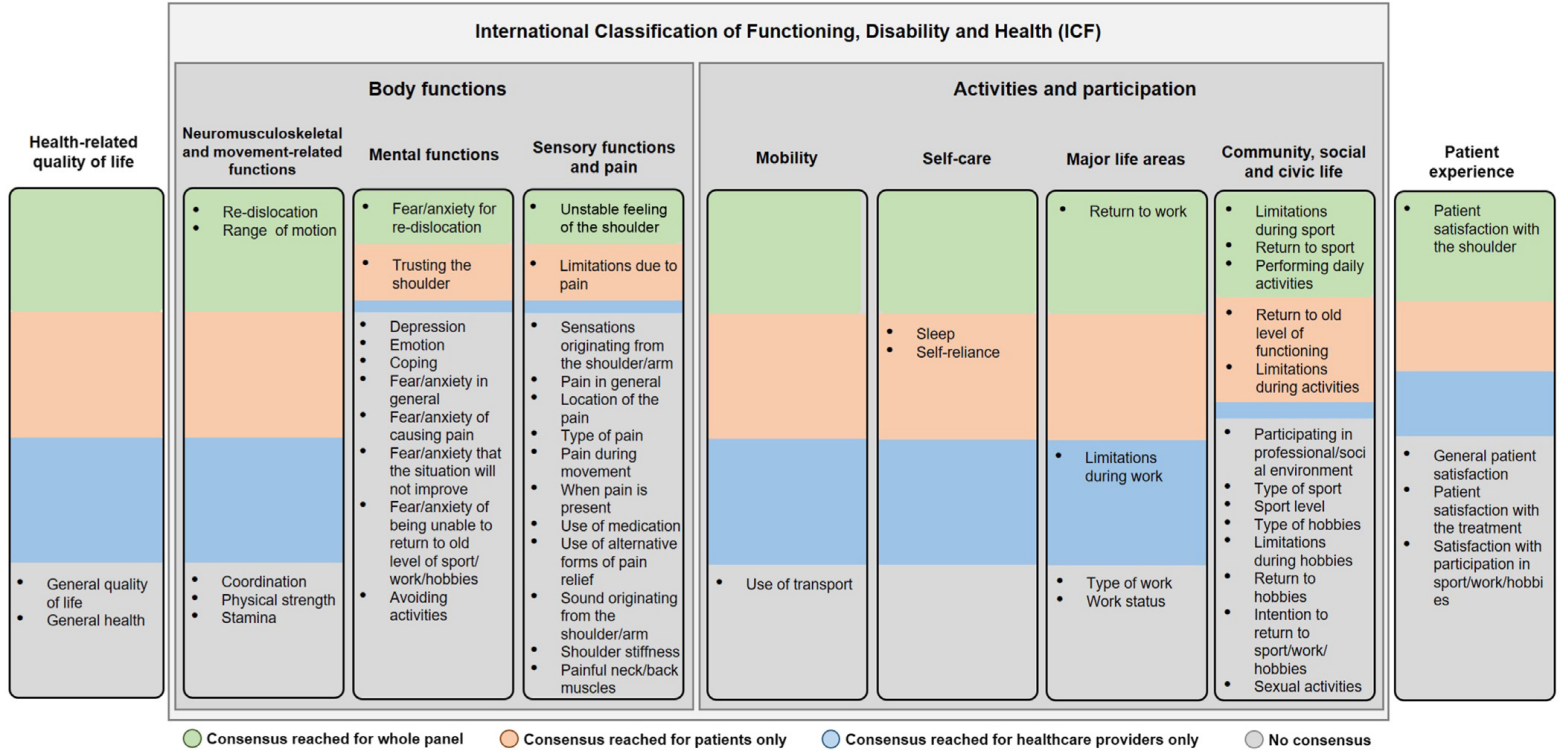
All items that were identified in round one were classified according to the International Classification of Functioning, Disability and Health (ICF) with the additional categories “health-related quality of life” and “patient experience.” The items are subdivided with colors, which indicate if the items reached consensus in round three for the entire panel *(green)*, only the patients (orange), only the healthcare providers *(blue)*, and if there was no consensus *(gray)*.

The search resulted in four systematic reviews on PROM used in shoulder instability research.^6^^,^^17^^,^^19^^,^^26^ These reviews included the following PROMs that were used: WOSI, American Shoulder and Elbow Surgeons score, University of California Los Angeles score, Disability of Arm, Shoulder and Hand, Simple Shoulder Test, Subjective Shoulder Value, Single Assessment Numeric Evaluation, Short Form-12 and 36, Oxford Shoulder Instability Score, Melbourne Instability Shoulder Score and Oxford Shoulder Score. A total of 48 items could be identified based on these PROMs. The panel added 6 additional items in the first round, which resulted in a total of 54 items to be evaluated in round two and three (Fig. 1).

### Second round

**Figure 2:**
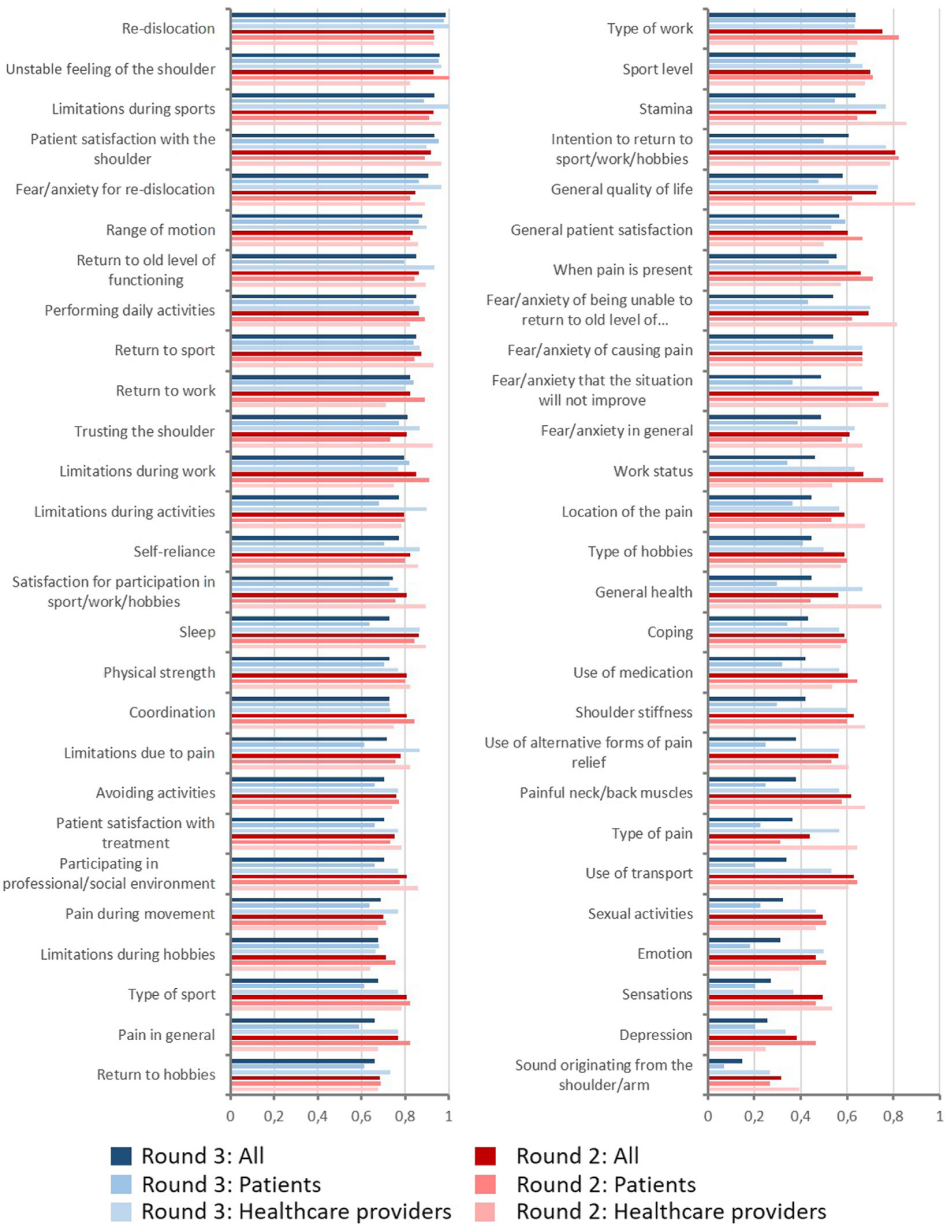
Each item is presented here with the percentage of the panel that gave the item a score of 7 or higher during round two *(red)* and three *(blue)*. The figure also shows the results of the healthcare providers and patients separately.

The panel reached consensus on 20 items that should be included in a questionnaire in round two, comprising limitations during sport (93%), unstable feeling of the shoulder (93%), re-dislocation (93%), patient satisfaction with the shoulder (92%), return to sport (86%), return to old level of functioning (86%), sleep (86%), performing daily activities (85%), limitations during work (85%), fear/anxiety for re-dislocation (85%), range of motion (84%), self-reliance (82%), return to work (82%), intention to return to sport/work/hobby (81%), satisfaction with participation in sport/work/hobbies (81%), type of sport (81%), shoulder strength (81%), coordination of the shoulder (81%), participating in professional/social environment (81%), and trusting the shoulder (81%; Fig. 2). Items that reached a consensus in the patient group and not in the healthcare provider group included trusting the shoulder, quality of life in general, stamina, limitations due to pain, and fear/anxiety of being unable to return to old level of sport/work/hobbies. Items that reached a consensus in the healthcare provider group and not in the patient group included limitations during work, return to work, type of sport, type of work, intention to return to sport/work/hobbies, and pain in general. None of the items reached a consensus that they should never be used in a questionnaire.

### Third round

After seeing the summary of round two, the panel reached consensus on 11 items that should be included in a questionnaire for shoulder instability research in round 3 (Table II). These comprised re-dislocation (99%), unstable feeling of the shoulder (96%), limitations during sport (93%), patient satisfaction with the shoulder (93%), fear/anxiety for re-dislocation (91%), range of motion (88%), return to old level of functioning (85%), performing daily activities (85%), return to sport (82%), return to work (82%), and trusting the shoulder (81%; Fig. 2). Items that reached a consensus in the patient group and not in the healthcare provider group included return to old level of functioning, trusting the shoulder, limitations during activities, self-reliance, sleep, and limitations due to pain (Fig. 1). An item that reached a consensus in the healthcare provider group and not in the patient group included limitations during work. None of the items reached a consensus that they should never be used in a questionnaire.

**Table II:** The 11 items that were considered important to include in a questionnaire.

Strong consensus (≥ 90%)	Consensus (≥ 80%)
Re-dislocation	Range of motion
Unstable feeling of the shoulder	Return to old level of functioning
Limitations during sports	Performing daily activities
Patient satisfaction with the shoulder	Return to sport
Fear/anxiety for re-dislocation	Return to work
	Trusting the shoulder

These items reached consensus following round three, meaning that ≥ 80% of the panel gave the item a score of 7 or higher on the 9-point Likert scale.

## Discussion

The goal of this study was to include patients and healthcare providers in a modified Delphi design to identify items that should be included in a questionnaire for shoulder instability research. These items can assist in developing a COS and facilitate standardized measurement in future trials and cohort studies. The panel reached a consensus on 11 items that should be included in a questionnaire for shoulder instability research. Re-dislocation, unstable feeling of the shoulder, limitations during sport, patient satisfaction with the shoulder, and fear/anxiety for re-dislocation reached a strong consensus. Range of motion, return to old level of functioning, performing daily activities, return to sport, return to work, and trusting the shoulder reached a consensus as well.

The performed study was not without limitations and therefore it should be interpreted in the light of the following remarks: First, there is no consensus on which methodology should be used to reach a consensus for a controversial topic. A Delphi design is structured, facilitates anonymous assessment with feedback from the panel and is therefore a preferred study design. It could also be argued that emergency doctors are not part of the follow-up of shoulder instability patients, but as they are active in shoulder instability research that includes these outcomes—therefore are stakeholders—and they are important in the inclusion of patients, they were included in this study. Second, the mean age of the included patients was relatively high for shoulder instability patients and it is unclear if the surgical patients experienced complications which could influence their opinion. Third, many items reached a consensus to be included in the questionnaire and none of the items reached a consensus for exclusion from the questionnaire. This could mean that all the factors carry some kind of importance or that more rounds needed to be organized to reach a consensus. Important items got a similar or slightly higher score in the third round compared to the second round, whereas items that were considered less important clearly got lower scorers in the third round. Fourth, the study was performed with Dutch healthcare providers and patients. An international panel is needed to identify which items should be included in the questionnaire for shoulder instability to be recognized internationally. Fifth, the study did not include stakeholders such as insurance companies or policymakers, which could have been a valuable addition for the market and societal point of view. The study also has some important strengths. First, to minimize the amount of bias in defining the items, they were presented to the entire panel for feedback. Second, most consensus statements overlook the patient’s perspective, but this study included the opinion of both patients and healthcare providers. Third, the guidelines by Diamond et al were used for the standardized design and reporting of a Delphi study.^5^

Re-dislocation, unstable feeling of the shoulder, limitations during sport and patient satisfaction with the shoulder reached a strong consensus and are common outcomes in shoulder instability research. However, there is no consensus on definition for these outcomes or how to measure them, hampering meta-analyses.^1^^,^^10^ For example, should a healthcare professional confirm a re-dislocation or can we acquire this information through a questionnaire as well? Fear/anxiety for re-dislocation also reached a strong consensus and is rarely used as outcome, even though it might be a valuable addition. Van Iersel et al already showed that patients consider this outcome to be important.^23^

To prevent inconsistencies in outcome measurement and outcome-reporting bias, the COMET initiative proposes to choose important health outcomes and define a COS.^7^^,^^28^ A systematic review by Whittle et al determined that the WOSI did not acquire information regarding all domains that are advised by the COMET handbook and lacks patient satisfaction.^26^^,^^28^ The current study showed that healthcare providers and patients agree that patient satisfaction with the shoulder is one of the most important domains to include as an outcome. Therefore, it would be recommended to add this outcome to the WOSI score during follow-up. New PROMs are still produced for shoulder instability research, such as the Shoulder Instability-Return to Sport after Injury (SI-RSI) score.^8^^,^^18^ Even though these scores can acquire valuable information, there is still no consensus on when to use which outcome. In addition, when multiple PROMs are used, some questions may be overlapping. Existing PROMs can be used as a basis to determine a COS for shoulder instability research.

The next step in designing a COS is to agree on a (small) set of outcomes for patients with shoulder instability that can be used on an international scale. Future research should therefore focus on an international consensus study that determines which items should be included in a COS for shoulder instability research and how these outcomes should be measured. This includes a consensus regarding which questions or PROM should be included in future studies and at which time points they should be measured. This consensus study should include patients and healthcare providers who work with shoulder instability. On a national scale, insurance companies and policymakers can be included as well to reach a consensus. Implementation specialist can assist in translating the results of this COS to a workable design that can be used in practice. Shoulder instability is characterized by heterogeneous outcomes and patient groups and therefore needs high-quality evidence with a large sample size to improve patient selection.^22^^,^^25^ A prospective cohort could provide these data; however, it can be challenging to determine which outcomes should be included in the questionnaires for patients. An unanimous COS can produce homogeneous data collection through trials and (prospective) cohort studies, which facilitates pooling data and comparing results on a national scale as well as internationally. It could also be possible that different types of instability, such as traumatic and atraumatic, warrant different COS.

## Conclusion

Healthcare providers and patients reached a consensus on 11 items that should be included in a questionnaire for shoulder instability research. These items can facilitate design and development of future clinical trials and form the basis for development of a COS.

## Acknowledgment

The authors thank all patients who contributed to the development of this manuscript.

## Disclaimers

Funding: This study was funded by 10.13039/501100001826ZonMw, the Dutch association for research and innovation in healthcare.

Conflicts of interest: D.F.P. van Deurzen is a paid instructor for Wright medical. A. van Noort is a consultant at LIMA (education and clinical research). The other authors, their immediate families, and any research foundation with which they are affiliated have not received any financial payments or other benefits from any commercial entity related to the subject of this article.
